# New Insight into HPts as Hubs in Poplar Cytokinin and Osmosensing Multistep Phosphorelays: Cytokinin Pathway Uses Specific HPts

**DOI:** 10.3390/plants8120591

**Published:** 2019-12-11

**Authors:** François Héricourt, Mélanie Larcher, Françoise Chefdor, Konstantinos Koudounas, Inês Carqueijeiro, Pamela Lemos Cruz, Vincent Courdavault, Mirai Tanigawa, Tatsuya Maeda, Christiane Depierreux, Frédéric Lamblin, Gaëlle Glévarec, Sabine Carpin

**Affiliations:** 1 LBLGC, University of Orléans, EA1207, INRA, USC1328, rue de Chartres, CEDEX 2, 45067 Orléans, France; francois.hericourt@univ-orleans.fr (F.H.); melanie.larcher@univ-orleans.fr (M.L.); francoise.chefdor@univ-orleans.fr (F.C.); christiane.depierreux@univ-orleans.fr (C.D.); frederic.lamblin@univ-orleans.fr (F.L.); 2 BBV, University of Tours, EA 2106, 31 Avenue Monge, 37200 Tours, France; koudounas@univ-tours.fr (K.K.); ines.carqueijeiro@univ-tours.fr (I.C.); pamela.lemos@univ-tours.fr (P.L.C.); vincent.courdavault@univ-tours.fr (V.C.); gaelle.glevarec@univ-tours.fr (G.G.); 3 Department of Biology, Hamamatsu University School of Medicine, 1-20-1 Handayama, Higashi-ku, Hamamatsu, Shizuoka 431-3192, Japan; tanigawa@hama-med.ac.jp (M.T.); tmaeda@hama-med.ac.jp (T.M.)

**Keywords:** HK, histidine-aspartate kinase, HPt, histidine phosphotransfer protein, CK, cytokinin, OS, osmosensing, MSP, multistep phosphorelay

## Abstract

We have previously identified proteins in poplar which belong to an osmosensing (OS) signaling pathway, called a multistep phosphorelay (MSP). The MSP comprises histidine-aspartate kinases (HK), which act as membrane receptors; histidine phosphotransfer (HPt) proteins, which act as phosphorelay proteins; and response regulators (RR), some of which act as transcription factors. In this study, we identified the HK proteins homologous to the Arabidopsis cytokinin (CK) receptors, which are first partners in the poplar cytokinin MSP, and focused on specificity of these two MSPs (CK and OS), which seem to share the same pool of HPt proteins. Firstly, we isolated five CK HKs from poplar which are homologous to Arabidopsis AHK2, AHK3, and AHK4, namely, HK2, HK3a, HK3b, HK4a, HK4b. These HKs were shown to be functional kinases, as observed in a functional complementation of a yeast HK deleted strain. Moreover, one of these HKs, HK4a, was shown to have kinase activity dependent on the presence of CK. Exhaustive interaction tests between these five CK HKs and the 10 HPts characterized in poplar were performed using two-hybrid and BiFC experiments. The resulting partnership was compared to that previously identified between putative osmosensors HK1a/1b and HPt proteins. Finally, in planta coexpression analysis of genes encoding these potential partners revealed that almost all HPts are coexpressed with CK HKs in four different poplar organs. Overall, these results allowed us to unravel the common and specific partnerships existing between OS and CK MSP in *Populus*.

## 1. Introduction

It has been known for a long time that the phytohormone cytokinin (CK) is involved in numerous physiological processes crucial to plant growth and development [1,2,3,4]. More recently, it became more and more obvious that this hormone also plays an important role in response to abiotic stresses such as drought, salinity, or cold [5,6,7,8]. Crosstalk between different signaling pathways seem to be a common feature in plant stress response and are beginning to be well documented [9,10,11,12,13]. Recently, researchers hypothesized the existance of a Yin-Yang balance between CK homeostasis and drought adaptation responses which modulates plant fitness and yields stability under drought [14]. At the cellular level, drought is perceived to be an osmotic constraint [15], and although the osmosensing (OS) and CK pathways have been studied, information on crosstalk between these two pathways remains elusive.

In *Arabidopsis thaliana*, the OS and CK pathways belong to a multistep phosphorelay (MSP) system derived from the bacterial two-component system [16]. In such a system, the membrane-bound histidine-aspartate kinase (HK) receptor senses its environment and autophosphorylates upon signal activation. This phosphate is further transmitted to a histidine phosphotransfer (HPt) protein which shuttles the signal to a response regulator (RR) in the nucleus, with some of the latter acting as transcription factors for targeted genes involved in the signaling pathway. In *A. thaliana,* the OS pathway seems to involve the protein AHK1 which belongs to the HK family [17]. This HK has the capacity to replace the function of the yeast osmosensor Sln1p in an osmodeficient yeast strain and could act as a positive regulator in osmotic stress signaling in planta [18,19]. Regarding the CK signaling pathway, three HKs containing the CK-binding domain (CHASE domain) have been isolated and characterized (reviewed in [4,20]). These CK HKs, namely, AHK2, AHK3, and AHK4 (CRE1), have been well studied and shown to interact with the four authentic HPt proteins, AHP1, AHP2, AHP3, and AHP5 [21]. However, it has been shown in this plant that AHK1 can only interact with one HPt protein, AHP2 [22], leading to the conclusion that AHP2 is therefore able to interact with either CK HK or OS HK.

In *Populus*, the OS pathway has been characterized, and two putative osmosensors, HK1a and HK1b, have been identified [23,24]. cDNAs encoding 10 HPts (HPt1–10) [25], 10 RRAs (RR1–8 and RR10–11) [26], and 9 RRBs (RR12–16, RR18–19, RR21–22) [27,28,29] have been isolated. A partnership between all these proteins has been characterized, thus defining an MSP for the HK1 signaling pathway in poplar [26].

An in silico analysis performed on different plant species for a study on the CK signaling evolution revealed the existence of five CK HKs in the genome of *Populus trichocarpa* [30]. More recently, another in silico analysis of CK signaling and homeostasis in two hardwood tree species also detected five CK HKs present in the poplar genome, whereas the peach genome only contains three CK HKs [31]. In poplar, this CK signaling pathway was shown to regulate the cambial development [32]. Although CK HK sequences have been identified in the poplar genome, they were never isolated nor analyzed in terms of their relationship with their HPt partners within the MSP.

Furthermore, it has been demonstrated in planta that AHP proteins constitute redundant positive regulators of CK signaling [33] and are also redundant negative regulators of drought stress response [34]. These findings illustrate the crosstalk between the CK and OS signaling pathways, leading the authors to question how a specific response to a specific signal can be achieved with a common pool of AHP proteins for both signaling pathways [35]. Only scarce information is available interconnecting the CK and OS signaling pathways in plants, and it has been established that only AHP2 interacts with both CK and OS HKs in Arabidopsis. On the other hand, no interaction network has been identified between these two pathways in poplar. One hypothesis could be that the genome duplication in poplar for HPt proteins could induce a functional specificity of duplicated HPts between the OS or CK pathways.

In the present work, the isolation and characterization of five new sequences encoding homologous poplar CK HKs (HK2, HK3a, HK3b, HK4a, and HK4b) are described. Their HK function has been established by the functional complementation of a *sln1Δ sho1Δ* yeast deletion mutant. Using a yeast two-hybrid (Y2H) system, all these HKs were tested with HPts, which allowed the partnership between CK HKs and HPts to be mapped. BiFC experiments were conducted to validate these Y2H results in planta. The resulting interactome was completed by analysing the gene coexpression of interacting HPts and CK HKs in *Populus* in four different organs. Overall, these data allowed us to compare this partnership with the one obtained previously for both HK1s and determine the specific and common HPt proteins involved in the CK and OS signaling pathways in *Populus* for the first time. Finally, hypotheses concerning our findings of HPt proteins as hubs in signaling specificity are discussed.

## 2. Results

### 2.1. Isolation of Five HKs from the Poplar Dorskamp Genotype

**Figure 1:**
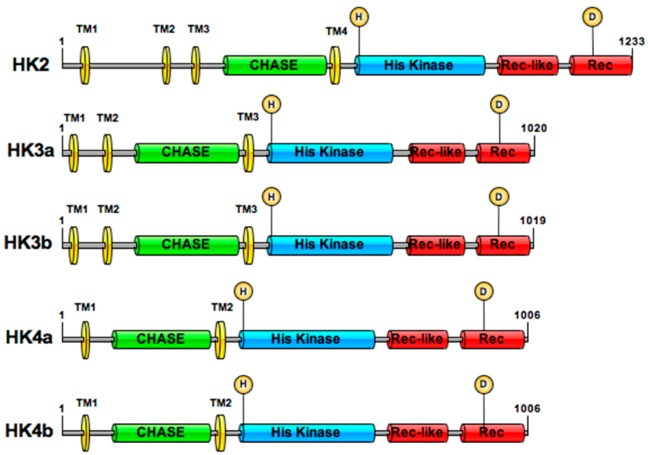
Cytokinin HKs in *Populus.* Protein organization of HK2 (accession No. LS484706), HK3a (accession No. LS484707), HK3b (accession No. LS484708), HK4a (accession No. LS484709), HK4b (accession No. LS484710) is shown. All domain lengths are based on the InterProScan analysis. TM: transmembrane domain; CHASE: Cytokinin binding domain; His Kinase: Histidine kinase domain; Rec: Receiver domain; Rec-like: Receiver-like domain. Predictive phosphorylation sites are shown with an H (Histidine) and D (Aspartate) in a yellow circle.

An in silico analysis of the *Populus trichocarpa* genome allowed five genes encoding homologous proteins to Arabidopsis CK HKs to be identified [30,31]. These five sequences were isolated from the poplar Dorskamp genotype and named HK2, HK3a, HK3b, HK4a, and HK4b due to their homology to Arabidopsis AHK2, AHK3, and AHK4 (Figure S1A). The deduced amino acid sequences of these five CDSs exhibit all the conserved domains and motifs which characterize the CK HKs (Figure 1). The overall homology of HK2 with HK3 pairs averages 57% and with HK4 pairs 54%, between HK3 pairs and HK4 pairs 62%. The homology within the HK3 and HK4 pairs averages 95%. This identification brings the number of non-ethylene HKs isolated from this genotype of poplar up to seven.

### 2.2. Isolated Poplar CK HK Sequences Are Functional HKs

**Figure 2:**
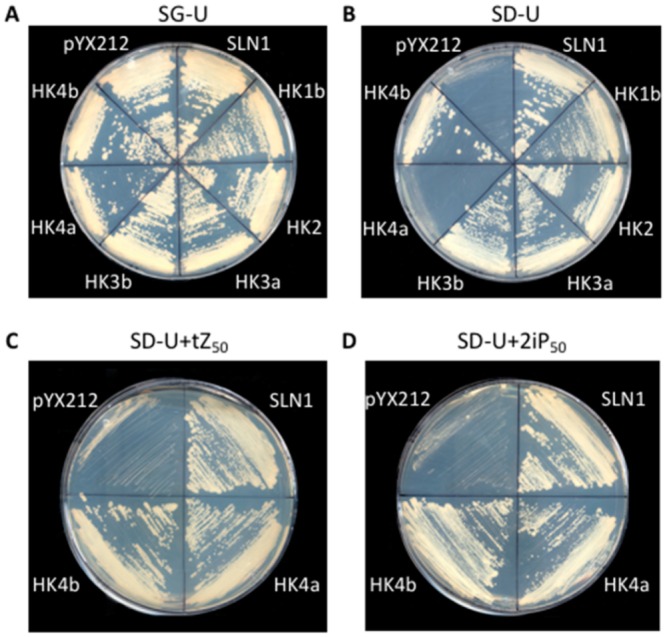
Functional complementation test with CK HKs. (**A**) Uracil-borne constructs (pYX212) were used to transform the MH179 mutant strain as indicated and the transformed yeasts were streaked onto galactose medium lacking uracil for growth control. (**B**) The same transformed yeasts were streaked onto glucose medium lacking uracil for a kinase activity test. (**C**) HK4a/4b transformant yeasts were streaked onto glucose medium lacking uracil supplemented with trans-zeatin (tZ) and (**D**) 2-isopentenyladenine (2iP) at 50 µM each.

In order to demonstrate the functional kinase activity for these poplar CK HKs, a *S*. *cerevisiae sln1Δ sho1Δ* deletion mutant strain (MH179) was transformed with plasmids carrying the full CDS of the five CK HKs. In parallel, the MH179 strain was transformed with a plasmid carrying the *SLN1* or *HK1b* cDNA, and with the empty pYX212 vector as positive and negative controls, respectively (Figure 2A). In this strain, *PTP2* expression under the control of an inducible galactose promoter allowed the survival of all transformants on galactose medium (SG-U, Figure 2A). On the glucose medium (SD-U), HK1b- and Sln1p-expressing yeasts exhibited normal growth whereas cells transformed with the empty vector had no growth (Figure 2B). All tested CK HKs showed a growth comparable to positive controls except for HK4a growth, which was comparable to the negative control (Figure 2B). Since the HK4a homologous protein in Arabidopsis (AHK4) was shown to complement this deletion yeast system only when cytokinin was supplemented in the medium [18], we tested this possibility with our HK4a protein. When the medium was supplemented with 50 µM of cytokinin trans-zeatin (tZ, Figure 2C) or isopentenyladenine (2iP, Figure 2D), HK4a was able to functionally complement the Sln1p HK deficiency. It is noteworthy that HK4b, which shares 95.8% homology with HK4a, has a contrasted behavior since it does not require the addition of cytokinin (Figure 2B–D). The same growth was observed when medium supplemented with only 5 µM of tZ or 2iP was used (data not shown). These results showed that all CK HKs present a functional kinase activity in yeast, and that this function is necessary for the complementation and recovery of the lethal phenotype of the *sln1Δ sho1Δ* yeast strain. Furthermore, in the case of HK4a, this functional kinase activity was dependent on the addition of cytokinin.

### 2.3. Identification of HPt Partners of CK HKs in Y2H Assay

A global yeast two-hybrid analysis was conducted in order to study interactions between the five isolated CK HKs and the 10 poplar HPts. Since HPt expression in the bait configuration led to a strong autoactivation, these proteins were tested in the prey configuration. Consequently, CK HKs were tested in the bait configuration by expressing their receiver domain (RD), which is the minimal interaction domain.

**Figure 3:**
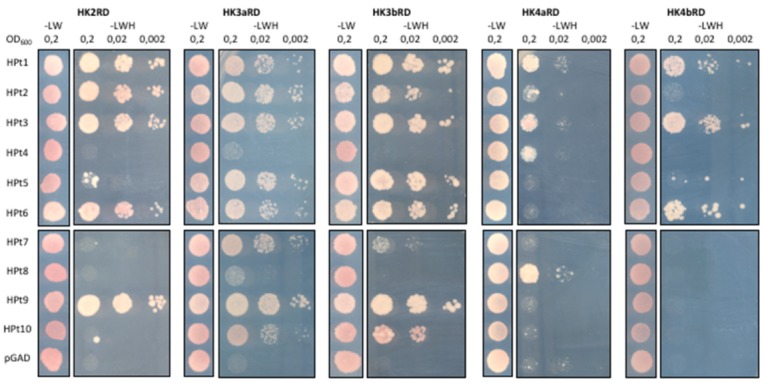
Y2H interactions between CK HKs and HPts. Receiver domains (RD) of HK2, HK3a, HK3b, HK4a, HK4b were tested for interaction with all HPt proteins in the yeast two-hybrid system. Cotransformed yeasts were spotted onto nonselective medium (-LW) for growth control and three dilutions were spotted onto selective medium (-LWH) for interaction test. Empty prey vector (pGAD) was used as negative control.

This interaction analysis revealed different interaction patterns for the five receptors (Figure 3). HK2 was able to strongly interact with five HPts (HPt1, 2, 3, 6, 9) and presented no or weak interaction with the five others (HPt4, 5, 7, 8, 10). We observed a very similar interaction pattern for HK3a and HK3b, which interacted with all HPts, except HPt4 and 8 (at least for HK3b). On the contrary, HK4a and HK4b interacted with very few HPts and displayed a contrasting pattern of interaction partners. Notably, only HK4a was able to interact with HPt4 and 8, along with HPt1, 2, 6, whereas HK4b was able to interact with only three HPts (HPt1, 3, and 6). Therefore, some HPts seem to interact preferentially with the two HK3s, such as HPt5, 7, and 10, and some others seem to interact preferentially with HK4a, such as HPt4 and 8. However, some HPts are common to all CK HKs, such as HPt1 and 3. This interaction analysis also allowed us to score the interactions by comparing the level of growth in each dilution between all CK HKs/HPts combinations (Table S1A).

### 2.4. Validation of HPt Partners of CK HKs in Planta

**Figure 4:**
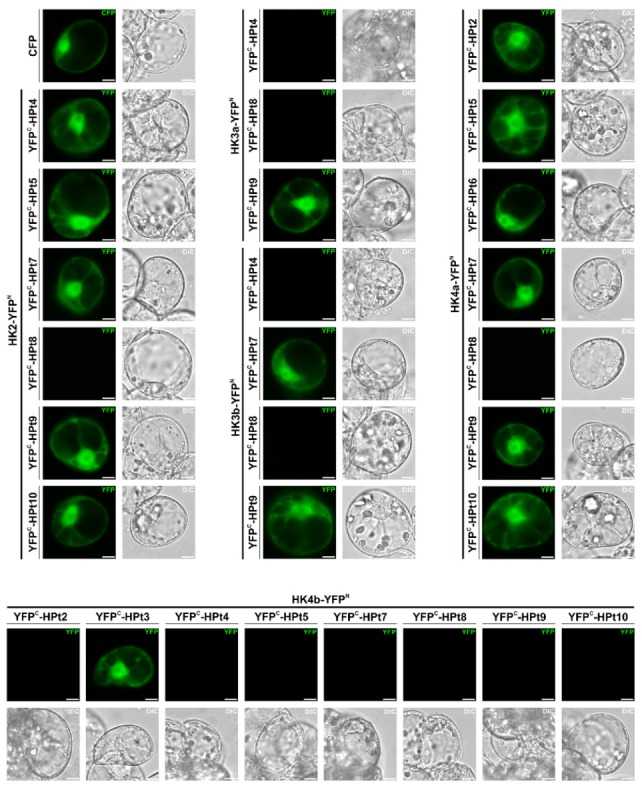
BiFC interactions between CK HKs and HPts. *Catharanthus roseus* cells were transformed with HK-YFP^N^ constructs in combination with YFP^C^-HPt constructs. Interaction between proteins reconstitutes a functional YFP resulting in a fluorescent signal. The CFP fluorescence corresponds to the nucleo-cytosolic marker. The morphology is observed with differential interference contrast (DIC) microscopy. Bar: 10 µm.

In order to confirm the lack of or the presence of weak interactions detected in Y2H assays, BiFC assays were conducted between all five CK HKs and different HPt proteins in planta. HKs were fused to the N-terminal fragment of yellow fluorescent protein (YFP^N^) and HPts to its C-terminal fragment (YFP^C^), to produce HK-YFP^N^ and YFP^C^-HPt fusion proteins. Interactions with a strong signal in the Y2H assay were used as positive controls (e.g., HK2/HPt9) and interactions with no signal were used as negative controls (e.g., HK4b/HPt10). The use of an RD for each HK accounts for the diffuse signal corresponding to a cytosolic localization. This BiFC approach was corroborated by some interactions observed in Y2H tests but not all of them (Figure 4). As shown in Figure 4, all tested HPts were able to interact with HK2, with the exception of HPt8. The results obtained for HK3a/3b showed an interaction with HPt7 and HPt9 but not with HPt4 and HPt8. The same results as those observed for HK2 were obtained with HK4a, with all tested HPts being able to interact, with the exception of HPt8. Conversely, apart from HPt3, HK4b was not able to show a positive YFP signal with all tested HPts.

When these BiFC results were compared with those obtained in the Y2H assay (Figure 3), we were able to validate the interaction of HK2 with HPt5 and HPt9, and the lack of interaction with HPt8, and to show undetected interactions with HPt4, HPt7, and HPt10. Concerning HK3a/3b, we confirmed the results observed in the Y2H assays, with the exception of HPt8, which was unable to interact in this system. This BiFC approach allowed us to confirm the interaction of HK4a with HPt2 and also to detect the interaction with HPt5, HPt6, HPt7, HPt9, and HPt10 that were not detected in the Y2H system. On the contrary, the interaction between HK4a and HPt8 was not confirmed. Finally, HK4b presented a contrasting pattern compared to HK4a, interacting with hardly any HPts, which confirmed the results from the Y2H assays.

### 2.5. Expression of CK HK and HPt Genes

**Figure 5:**
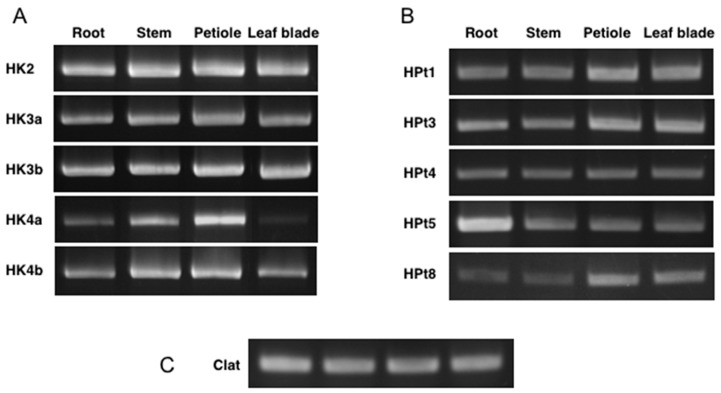
Expression profile of CK HKs and HPts using RT-PCR. RNAs isolated from the roots, stems, petioles, and leaf blades were reverse transcribed and used as template for PCR amplification of CK HKs (**A**) and HPts (**B**) as indicated. The expression profile of *Clathrin* (Clat) was used as control expression gene (**C**).

In order to substantiate the characterized interactions, the potential coexpression of CK HKs with HPts was studied in planta. Expression was monitored in the roots, stems, petioles, and leaf blades with reverse transcriptase-polymerase chain reaction (RT-PCR) in control conditions (Figure 5). *Clathrin* gene (Clat) was used as an amplification control (Figure 5C). Results showed that all CK HKs have a constitutive expression since they were detected in all tested organs (Figure 5A). *HK2*, *HK3a*, and *HK3b* seem to be expressed homogeneously in the four organs. *HK4a* and *HK4b*, however, seem to be more expressed in the stem and the petiole, especially for *HK4a*, which is poorly detected in the roots and even more poorly in the leaf blades. In previous experiments, the expression profiles observed for HPt2 and HPt9 were homogeneous in these four organs and the expression of HPt7 was observed in all organs, but predominantly in the roots [25]. On the other hand, the expression of HPt10 was strictly restricted to the leaf blades, and no expression was detected for HPt6 in these four organs [25]. For the five other HPt genes, the results showed that they are all expressed in the four organs (Figure 5B). HPt4 seems to be homogeneously expressed while HPt1, HPt3, and HPt8 seem to be preferentially expressed in the petioles and leaves. Finally, HPt5 presents a clearly predominant expression in the roots. Altogether, these data indicated that the CK HKs are coexpressed along with nine HPts in the plants and are restricted to the leaf blades in the case of HPt10.

## 3. Discussion

In the context of water deficit, plants have to adapt their growth development to this stress condition and physiological adjustments are required to allow survival. Thus, a subtle balance between normal physiological processes and stress response is expected to occur. Recent findings revealed that cytokinin and its signaling pathway can act as negative regulators of plant drought tolerance [8,14]. In the environmental context of increasing drought periods, it is highly important to understand how plants are able to control CK homeostasis via the CK signaling pathway to preserve growth while tolerating drought. This notion leads to the hypothesis of a Yin-Yang relationship between CK homeostasis and plant acclimation/adaption to drought [14]. At the same time, an OS signaling pathway has been identified in Arabidopsis and is controlled by the AHK1 histidine-aspartate kinase [17,18,19]. In this hypothesis, CK and OS signaling pathways seem to have an antagonist role. Accordingly, it has been demonstrated in Arabidopsis that AHP proteins constitute redundant positive regulators of CK signaling [33], and are also redundant negative regulators of the drought stress response [34]. These observations raised the question of how a specific response to a specific signal can be achieved with a common pool of AHP proteins for different signaling pathways [35]. Generally, almost all genes belonging to these signaling pathways, especially HPts and RRs, are duplicated. It has now been established that gene duplication is an important process in plant adaptation to abiotic or biotic changing environments and that paralogs could acquire novel functions that contribute to better plant adaptation [36]. One hypothesis about the signaling specificity between growth or tolerance could be based on the genome duplication for HPt genes which could induce functional specificity for duplicated HPt proteins between CK or OS pathways.

In previous studies, we identified HPt and RR proteins which could participate (along with both HK1s) in the poplar OS signaling pathway [23,24,25,26,27,28,29]. Thus, in order to address the question of HPt specificity between CK and OS signaling pathways in poplar, we characterized the specific partnership of CK HKs.

For this purpose, we isolated five sequences which are homologous to Arabidopsis CK HKs in the poplar Dorskamp genotype. As it was previously observed, the evolutionary history of the CK HK genes led to the duplication of these genes in poplar, with the exception of HK2 [30]. This phenomenon concerns thousands of genes in poplar due to a whole-genome duplication event in the *Salicaceae* family [37]. The same duplicated genes are encountered in the tree species *Malus domestica* where five homologous genes are present [38]. On the contrary, only three genes homologous to the three Arabidopsis CK HKs were found in the tree *Prunus persica* [31]. The structure of poplar HK2, HK3a/*3b*, and HK4a/4b is typical of CK HK proteins described in numerous articles and summarized in different reviews [39,40,41]. The HK1a/1b proteins have a similar overall structure with an extracellular domain (ECD) between the two last transmembrane domains and two functional domains (His Kin and Rec) in the cytoplasmic part (Figure S1B). Nevertheless, differences can be observed in the ECD since there is no CHASE domain in either HK1 ECD, nor is there a Rec-like domain in the cytoplasmic part. This comparison shows that these proteins should have common mechanisms and partners, but they also have different signals, which implies specific partners too. Therefore, in order to determine the specificity of the signaling pathway, we investigated the partnership of all poplar CK HKs with all poplar HPt proteins to compare with the ones previously characterized for OS HKs [24,25].

First of all, we tested the capacity of these five poplar CK HKs to act as functional kinases in a functional complementation assay based on an osmodeficient mutant yeast strain. Since the characterization of MSP is involved in the osmosensing pathway in yeast [42,43], the yeast HK-deficient strains have been frequently used to test the functionality of HK proteins [17,18,24,25,38]. This experiment allowed us to show that HK2, HK3a/*3b*, and HK4a/4b are functional histidine kinases in this heterologous system and that HK4a, but not HK4b, presents a cytokinin-dependent activity. In Arabidopsis, the three CK HKs were tested in this yeast system and showed a similar result, with AHK2 and AHK3 being able to complement in a constitutive manner, but AHK4 only being functional upon the addition of CK [18]. In contrast, in apple trees, all CK HKs presented a strictly CK-dependent activity, with the exception of MdHK3b, and to a lesser extent MdHK3a [38]. As a common feature in these three different plant models, AHK4-like receptors seem to act in a similar way but HK2 and HK3 behave in a variable way according to plant species. Although apple tree and poplar are both woody plants, discrepancies between these two plants have already been observed for another HK protein. Indeed, a phylogenic tree built with AHK1-like sequences from numerous plant species showed a clear separation between these two species with only one AHK1-like gene in apple tree and two in poplar [24]. It is noteworthy that poplar HK4a and HK4b have contrasting behavior in this test regarding the CK dependence (Figure 2). This difference reinforces the idea that duplicated genes can generate paralogous proteins with divergent functions, as was shown for HK1a/1b in poplar [24].

To further characterize these poplar CK HKs, phenotype rescue experiments would be of great interest. Recently, Persinova et al. [44] developed a one-step hypocotyl explant assay to demonstrate that CKs inhibit auxin-induced organ establishment and mediate the early disorganisation of organ primordia to further switch the primordia identity. The authors made use of double *ahk* mutant lines of Arabidopsis to show the role of each AHK in this reprogramming process. Such a reporter system could provide a useful means of validating the biological activity of each poplar CK HK. Their expression in these double mutant lines, as well as the triple ahk2/ahk3/ahk4 mutant line used in Riefler et al. [45], could demonstrate their functional properties in planta. Moreover, a test of sensing of the different CKs would be of interest to complete the characterization of these poplar CK HKs, especially because it was shown recently that not only isoprenoid but also aromatic CKs are widespread amongst *Populus* species [46]. A very recent article presents a characterization of poplar CK HKs and their sensing to different CKs [47]. All together, these different approaches would allow a detailled description of these poplar CK HKs and could serve for a comparative study on CKs content and sensing variations in a context of drought stress.

In order to decipher the specificity of both CK and OS signaling pathways in poplar, we investigated the interaction network between CK HKs and HPts by a global Y2H analysis (Figure 3). The results showed that HK2 is able to interact with all HPts except for HPt8. HK3a/3b have a very similar interaction profile with only one notable difference regarding HPt8 interaction. On the contrary, HK4a/4b have highly contrasting profiles of interaction with HPt proteins. With the exception of HPt1 and HPt3, these two paralogous receptors present a completely opposite HPts preference. Furthermore, all weak or absence of interactions detected in the yeast system were tested in a plant system to support these results. The compilation of both interaction assays allowed us to confirm most of the interactions and also to detect new ones (Table S1A). The fact that all Y2H interactions were not confirmed in BiFC assays is somehow inherent to the system. These two techniques are based on fusion proteins and the reconstitution of a functional reporter protein. Any steric hindrance due to the 3D structures of fusion proteins, preventing this reconstitution, leads to false negative results. This issue has already been encountered in other protein–protein interaction studies on MSP proteins [27,41,48]. It is intriguing to note that HPt4 and HPt8 in poplar present weak interaction with CK HK receptors (this study) and no interaction with OS HKs [24,25], and are both AHP4-like proteins. In a study on CKI1 in Arabidopsis, it was shown that this non-ethylene HK could interact with all AHP proteins except AHP4 [41]. The same result was observed with another HK protein, AHK5, where AHP4 was the only AHP protein unable to interact with it [49]. To our knowledge, the interaction of this AHP protein with CK HKs has not been tested in Arabidopsis. However, this HPt protein was shown to be active in different physiological processes [50,51] and is homogenously expressed in poplar. These observations raise the question of how AHP4 can mediate its activity with almost no interaction with HK receptors. Interestingly, three HPts in poplar are grouped with AHP4 in a phylogenetic tree, HPt8/10 pair and HPt4 [25]. This diversity of AHP4 homologous proteins in poplar (HPt4, HPt8, and HPt10) could be indicative of the different behavior between poplar and Arabidopsis in terms of the use of the AHP4-like proteins.

The interaction analysis was further completed with a coexpression analysis in order to make an arguement for a genuine interaction. To be biologically relevant, a protein–protein interaction has to take place in a tissue or organ where both proteins are coexpressed. This expression analysis allowed us to demonstrate that all CK HKs are effectively coexpressed with all HPts, with the exception of HPt6. Recently, CK HKs expression has been analyzed in another tree species, *Malus domestica* [38]. According to our results, apple tree CK HKs MdHK2, MdHK3b, MdHK4a, and MdHK4b are expressed in the roots, stems, and leaves in a similar way to what we observed in poplar. In contrast, MdHK3a was mainly expressed in the roots, which is not the case for poplar. In Arabidopsis, the AHK2 and AHK4 expression profiles are completely different from our poplar results, but AHK3 presents an expression profile in the roots, stems, and leaves [52] similar to that of our poplar HK3s results. A very recent study on CK signaling pathways in six legume species revealed expression profiles for CK HKs in *Medicago truncatula* [53] in total accordance with those of poplar. These expression comparisons between different plant species illustrate the importance of studying different plant models since different patterns are encountered depending on the plant species.

**Figure 6:**
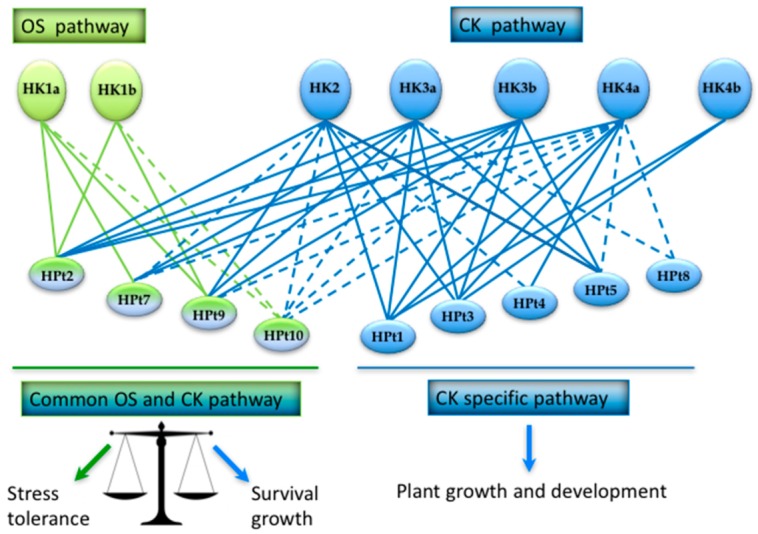
Interconnection between osmosensing and cytokinin pathways. The diagram represents the set of HPt proteins and their relationships with both osmosensing and cytokinin HKs. Continuous lines represent strong or confirmed interactions (Y2H and BiFC) and dashed lines represent interactions observed in only one system (Y2H or BiFC) or in a tissue-specific manner (HPt10 in leaf). HPts specific to cytokinin HKs are shown in blue and HPts common to all HKs are shown in green and blue.

All together, these data allowed us to compare the CK HKs partnership with the one previously characterized for OS HKs [24,25]. This comparison (Table S1) gave us the opportunity to identify HPt proteins which are common to both OS and CK HKs and those which are specific to CK HKs. Figure 6 presents the set of all HKs/HPts interactions characterized in poplar MSP, thus illustrating the fact that HPt proteins can serve as hubs in different signaling pathways. In this regard, we identified HPt2, HPt7, HPt9, and HPt10 as common HPts between OS and CK MSP and HPt1, HPt3, HPt4, HPt5, and HPt8 as specific HPts to CK MSP.

These findings in poplar are in agreement with the situation observed in Arabidopsis where some HPts are common to both MSPs and some others specific to the CK MSP. Among the five AHPs, only AHP1, 2, 3 were tested for their interaction with both OS and CK HKs. Although all three AHPs can interact with all the CK HKs [21], only AHP2 is able to interact with OS AHK1 [22]. Therefore, AHP2 constitutes the common HPt between both OS and CK HKs, and AHP1 and AHP3 are specific HPts to CK HKs. It remains to be determined which pool of HPts the two last AHPs (AHP4 and AHP5) belong to.

The notion of HPts as hub proteins is a concept that emerged a few years ago with the growing amount of MSP interaction network identification [21,54,55]. As integrative proteins in MSP are able to interact with upstream and downstream partners, it was proposed that HPt proteins act as hubs within the CK signaling pathway in Arabidopsis [21]. This idea was slightly modulated by Pekárová et al. [54] who argued that HPts are not unspecific signaling hubs but can achieve a certain level of interaction specificity. In Arabidopsis, numerous examples have unveiled the crosstalk between CK signaling and other pathways. Very recently, it was shown that CK and ethylene signaling pathways are interconnected via interactions between HPts, and control root apical meristem size through MSP activation [56]. The same pool of HPt proteins is also involved in the female gametophyte development and vegetative growth through the CK-independent HK CKI1 MSP [41,57,58]. The hydrogen peroxide-induced and ethylene-induced stomatal closure is also controlled by HPt proteins phosphorylated by AHK5 [59]. In rice, the mapping of the MSP network led the authors to conclude that OsPHPs could function as interaction hubs, indicating a crosstalk between signaling pathways [55].

One hypothesis on signal specificity was based on the genome duplication in poplar, which could induce functional specificity of duplicated HPts between OS or CK pathways. Our results showed that the HPt1/3 pair which groups with HPt5 was specifically dedicated to the CK pathway. On the contrary, HPt2/6 and HPt7/9 pairs, belonging to the same phylogenetic group, were able to interconnect both OS and CK signaling pathways. Only the duplicated HPt8/10 led to a divergence with HPt8 as CK-specific, and HPt10 as interconnection protein between both signaling pathways. Our results suggest that the HPt gene duplication does not mediate the signal specificity and no HPt protein is OS-specific in poplar. Another hypothesis, involving direct interaction between OS and CK HKs, could be proposed. To our knowledge, heterodimerization assays between OS and CK HKs have never been performed. The only heterodimerization assays reported in the literature concern interaction tests within the CK HKs family in Arabidopsis [21,60] and apple tree [38] or within the ethylene HKs family [61]. With a Y2H system, Dortay et al. [21] revealed only two heterodimerizations: AHK2/AHK3 and AHK3/AHK4. These results were confirmed in an in vitro GST pull-down assay and AHK3/AHK4 heterodimer was further confirmed in a BiFC assay [60]. On the contrary, almost all CK HKs from apple tree were able to heterodimerize with each other in a BiFC experiment, although it was not confirmed by any other methods [38]. Likewise, all ethylene receptors can form heterodimers with each other [61]. Thus, we tested the possibility of heterodimerization between OS and CK HKs by testing interactions between HK1a/1b and CK HK proteins in the Y2H system. The results showed no interaction for all tested combinations of heterodimers (Figure S2). This observation suggests that interconnection between the two signaling pathways may not be mediated by direct HK interactions. However, a validation of these results must be made through a BiFC approach to rule out this hypothesis. Therefore, our results on heterodimerization assays between poplar OS and CK HKs constitute novel information, albeit preliminary.

All these data emphasize the interconnection between MSP pathways, and the antagonist role that OS and CK signaling could play in response to drought stress. In accordance with this idea of a balance between these two pathways, we have identified the specific and common partners that could participate in this equilibrium. We can hypothesize that common HPts could be the pool of HPts interacting with CK HKs in normal growth conditions, and are thus unavailable for interaction with OS HKs. In a drought stress condition, a switch between HPt pools could occur: the specific HPt pool would assume the CK signaling in this stress context and the common HPt pool would be available for OS HKs signaling. Likewise, this switch could be triggered by the fine-tuning of cytokinin levels during a drought in key organs involved in molecular responses [14] and the cytokinin decrease could favor the OS pathway using common HPts.

These findings shed some light on signaling specificity between the two CK and OS pathways in poplar. As proposed by Pekárová et al. [54], more structural data is needed about MSP partners and mainly HPt proteins in order to clearly understand how plants are able to discriminate the signal using a common pool of HPt proteins. Furthermore, a more precise tissue localization of each partner could help to answer this question. Elucidation of the mechanisms underlying these crosstalks should give important information on the plant fine-tuning response to environmental cues.

## 4. Materials and Methods

### 4.1. Isolation of CK HK CDSs

Genes of CK HKs were identified in the genome of *Populus trichocarpa* (JGI Phytozome v12, Poplar v3.0) using the results of a Blast analysis with Arabidopsis sequences of AHK2, AHK3, and AHK4. Primer pairs corresponding to nucleotides just upstream and downstream of the start and stop codons, respectively, were designed to amplify full-length CDS of each CK HK (Table S2). PCRs were performed with primers at a final concentration of 0.4 µM and Q5 HF polymerase (New England Biolabs) on *Populus deltoides* (Bartr.) Marsh × *P. nigra* L. Dorskamp roots, stems, petioles, or leaf blades cDNAs. The resulting amplification sequences were cloned into the pGEM-T easy vector (Promega) and nucleotide sequences were analyzed by sequencing. Protein sequences were analyzed on the InterProScan website [62] and illustrated with IBS software [63].

### 4.2. Complementation Analysis of the sln1Δ sho1Δ Deletion Mutant MH179

The full CDSs of CK HKs were amplified by PCR and inserted into the pYX212 linearized vector (*Nco*I-*Xho*I digested) by homologous recombination in bacteria (InFusion kit, Clontech) for, HK3a, and HK3b or directly in yeast [64] for HK4a and HK4b (Table S2).

The pPD2133 plasmid expressing the Sln1p osmosensor was used as a positive control and the empty vector pYX212 as a negative control. The yeast strain MH179 (*ura3 leu2 his3 sln1::LEU2 sho1::TRP1* + pGP22, with pGP22: *GALp-PTP2/pRS413 (HIS3, CEN)*) was used for transformation. Yeast cells were grown on galactose-containing medium lacking uracil (SG-U) for the transformation control and streaked onto SD-U medium for four days at 30 °C for kinase activity complementation tests. For the HK4a test, cytokinins trans-zeatin (tZ) and 2-isopentenyladenine (2iP) were added to the SD-U medium to a concentration of 50 µM.

### 4.3. Yeast Two-Hybrid Tests

The two-hybrid assays were performed using a Gal4 DNA-binding domain encoding bait vector (pGBKT7, Clontech) and a Gal4 activation domain encoding prey vector (pGADT7, Clontech). The HK RD and CP sequences were cloned into the pGBKT7 vector as *Eco*RI-*Sal*I fragments (HK2 and HK4b), or as *Xma*I-*Sal*I fragments (HK3a, HK3b, HK4a) (Table S2). The HK CP sequences were cloned into the pGADT7 vector as *Eco*RI-*Xho*I fragments (HK1a and HK1b). HPt CDSs were cloned into the pGADT7 vector as described in [24].

The yeast strain PJ696 (*MATa gal4∆ gal80∆ ade2-101 his3-200 leu2-3,112 trp1-901 ura3-52 met2::GAL7-lacZ LYS2::GAL1-HIS3 GAL2p-ADE2 cyh^r^2*) was used for transformations according to [65]. Cotransformed yeasts were spotted onto leucine–tryptophan-lacking medium (-LW) at an OD_600_ of 0.2 and onto leucine–tryptophan–histidine-lacking medium (-LWH) along with 10X and 100X dilutions. Growth was allowed for four days at 30 °C.

### 4.4. BiFC Assays

BiFC assays were conducted using the pSPYCE(MR) [66] and pSPYNE173 plasmids [67] which allow the expression of a protein fused to the C- or N-terminal of the split-yellow fluorescent protein (YFP) fragments, respectively. The cDNA of CK HK-RD was cloned into the *Spe*I site via homologous recombination (InFusion, Clontech) in frame with the N-terminal fragment of YFP in pSPYNE173 (Table S2). The coding sequences of HPts were cloned into the pSPYCE(MR) as previously described [24].

Transient transformation of *Catharanthus roseus* cells by particle bombardment and YFP imaging were performed according to [68] with adaptation for BiFC assays [69].

### 4.5. Expression of HK and HPt Genes

Cuttings of *Populus deltoides* (Bartr.) Marsh × *P. nigra* L., clone Dorskamp, grown in hydroponic conditions were used. After the collection of cuttings, the roots, stems, petioles, and leaf blades were then harvested and frozen in liquid nitrogen. Total RNAs were extracted using the NucleoSpin^®^ RNA Plant mini kit (Macherey-Nagel) according to the manufacturer’s protocol, and reverse transcription was done with the M-MuLV Reverse Transcriptase RNase H- (Finnzyme) using one µg of RNAs. Specific primers for each HK (Table S2) were used at 0.2 µM for amplification of 1 µg of cDNAs by PCR (35 cycles). For HPt amplification, 30 or 35 PCR cycles were used and *Clathrin* cDNA expression was used as an amplification control with 30 cycles.

## Supplementary Materials

The following are available online at https://www.mdpi.com/2223-7747/8/12/591/s1, Figure S1: CK HKs from Arabidopsis and poplar and OS HKs organization, Figure S2: Y2H heterodimerization assay of HK1s/CK HKs, Table S1: Summary of interactions between HKs and HPts, Table S2: Primers used in this study.
