# Supplementary figures and images for: New Insight into HPts as Hubs in Poplar Cytokinin and Osmosensing Multistep Phosphorelays: Cytokinin Pathway Uses Specific HPts
Source: Plants (Basel). 2019 Dec 11;8(12):591. doi: 10.3390/plants8120591 (PMC6963366; doi:10.3390/plants8120591)

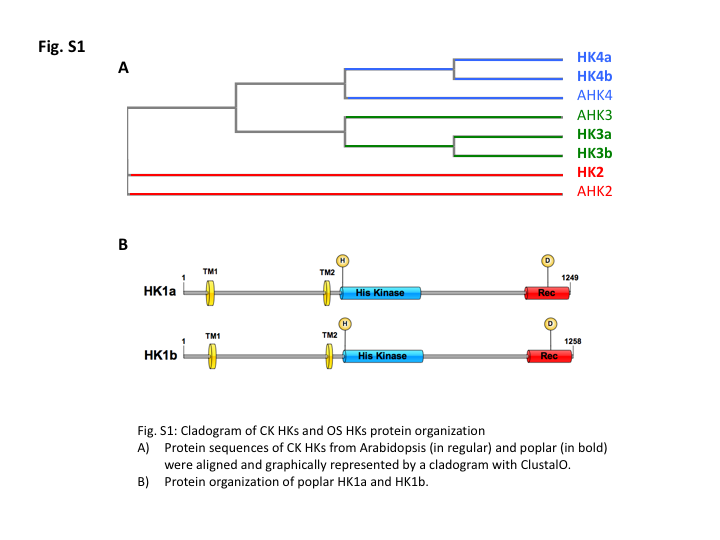

Supplement: Supplementary file 1 [file plants-08-00591-s001.zip › plants-661824-proof01-supplementary/Fig S1.png]

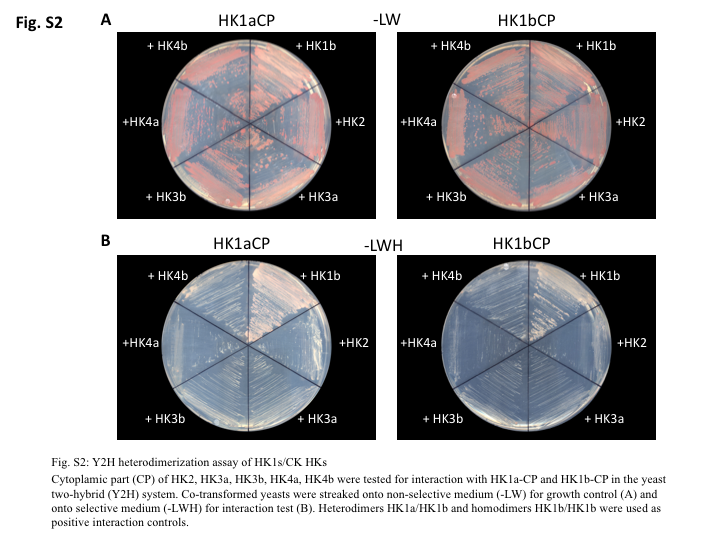

Supplement: Supplementary file 1 [file plants-08-00591-s001.zip › plants-661824-proof01-supplementary/Fig S2.png]

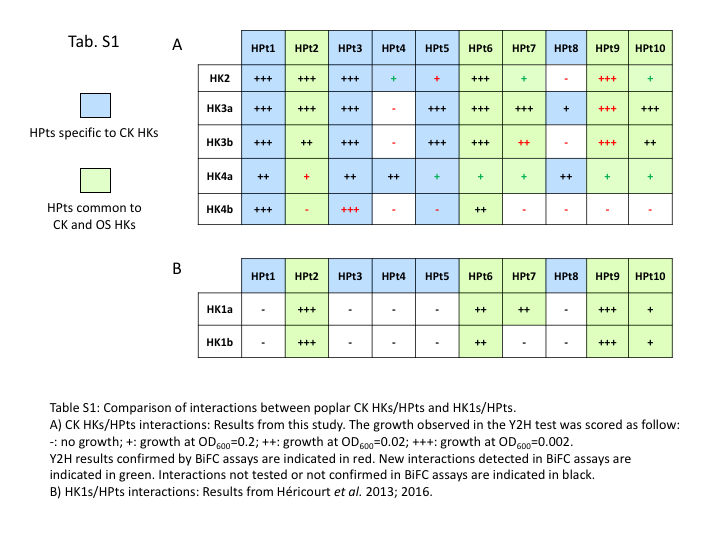

Supplement: Supplementary file 1 [file plants-08-00591-s001.zip › plants-661824-proof01-supplementary/Tab S1.png]
